# Innovating transfusion training: a BOPPPS-based blended learning model to enhance nursing interns’ specialized competency

**DOI:** 10.3389/fmed.2026.1759418

**Published:** 2026-02-10

**Authors:** Ningting Xiao, Chao Liu, Min Mao, Juan Huang, Yin Huang, Haiyan Wu, Wen Wang

**Affiliations:** 1 Department of Hematology, Sichuan Provincial People's Hospital, University of Electronic Science and Technology of China, Chengdu, Sichuan, China; 2 Department of Nursing, Sichuan Provincial People's Hospital, University of Electronic Science and Technology of China, Chengdu, Sichuan, China

**Keywords:** BOPPPS model, nursing education, nursing interns, self-directed learning skills, specialty training

## Abstract

**Introduction:**

Traditional clinical internship training for nursing interns’ transfusion skills is commonly characterized by unstructured design and insufficient personalized guidance, which leads to inconsistent learning outcomes and inadequate self-directed learning abilities. High-stakes clinical procedures such as transfusion lack standardized, targeted training in the conventional rotational internship system, creating persistent educational gaps for nursing interns.

**Methods:**

A quasi-experimental study was conducted between October and December 2024 among 78 nursing interns. Participants were allocated to a control group (*n* = 38) receiving traditional transfusion skills training, or an experimental group (*n* = 40) receiving blended specialty training based on the bridge-in, objective, pre-assessment, participatory learning, post-assessment, and summary (BOPPPS) framework. Outcome measures encompassed theoretical knowledge, transfusion operational skills, and self-directed learning (SDL) abilities, assessed using validated questionnaires (Cronbach’s *α* > 0.7) and standardized objective structured skill evaluations. Semi-structured interviews were performed among the experimental group and analyzed via thematic analysis. Quantitative data with normal distribution were analyzed using independent-samples t-tests, with a significance level defined as *p* < 0.05.

**Results:**

The experimental group exhibited significantly higher scores in theoretical knowledge (68.91 ± 10.53 vs. 60.47 ± 17.03, *p* = 0.010) and SDL abilities (105.88 ± 7.30 vs. 94.84 ± 8.17, *p* < 0.001) compared with the control group. No statistically significant between-group difference was detected in transfusion operational skills (experimental group: 90.35 ± 2.02; control group: 89.71 ± 1.94; *p* = 0.160). Thematic analysis of interview data identified three core themes: recognized deficiencies in clinical specialty practice, recommendations for training model optimization, and perceived learning benefits and competence improvements.

**Discussion:**

The BOPPPS-based blended specialty training model effectively ameliorates key shortcomings in traditional nursing internship education by significantly enhancing nursing interns’ theoretical mastery of transfusion and self-directed learning capabilities. The high engagement and learning motivation observed in this study support the model as a structured, replicable, and complementary educational strategy for the conventional rotational internship. Integrating this standardized training model can help address the lack of formalized procedural skill training for high-risk clinical practices, thereby promoting consistent and high-quality clinical competency development among nursing interns.

## 1 Introduction

Clinical internships represent a pivotal phase in nursing education because they bridge the gap between theoretical knowledge acquisition and clinical practice application while facilitating the professional socialization of nursing students into registered nurses (1). Despite their importance, the dominant pedagogical approach in clinical internship training remains a traditional, instructor-centered “transmission model” in many regions worldwide. This model is widely criticized for its limited capacity to cultivate students’ learning initiative, active engagement, and critical thinking skills (2–4). Compounding this issue, the conventional rotational internship system—typically structured around core clinical disciplines, including internal medicine, surgery, gynecology, and pediatrics—affords nursing interns only limited exposure to specialized nursing domains. Such insufficient specialty training not only creates gaps in targeted knowledge and skills but also impedes the development of comprehensive clinical competency—a critical outcome for safe and effective nursing practice.

Blood transfusion is a high-stakes, high-risk clinical intervention in which mismanagement can lead to severe, even life-threatening, adverse reactions. As such, proficiency in transfusion nursing demands a robust foundation of theoretical knowledge, meticulous procedural skills, and sound clinical judgment. However, within the compressed and overloaded schedule of traditional clinical rotations, nursing interns often receive fragmented, opportunistic, and unstandardized exposure to transfusion practices. The absence of systematic, structured, and guaranteed training in this critical specialty area not only compromises the development of interns’ transfusion nursing competence but also poses a tangible risk to patient safety in clinical settings.

The BOPPPS teaching model—a structured, learner-centered instructional framework comprising bridge-in, objective, pre-assessment, participatory learning, post-assessment, and summary phases—has demonstrated consistent efficacy in enhancing student motivation, engagement, and self-directed learning (SDL) abilities across diverse educational contexts, including nursing education (5–7). Despite its pedagogical strengths, the traditional offline implementation of the BOPPPS model is frequently constrained by temporal and spatial limitations, which hinder the establishment of timely, continuous feedback loops between instructors and students and limit the scalability of the intervention. Blended online-to-offline learning has emerged as a promising solution to these limitations by integrating the flexibility of online learning with the interactivity of in-person instruction (8). To address the well-recognized deficiencies in current transfusion nursing training and harness the synergistic potential of the BOPPPS framework and blended learning, this study aims to develop and implement a BOPPPS-based blended learning model tailored to the specialized context of transfusion nursing education for clinical interns. Specifically, it seeks to evaluate the effectiveness of this novel model in improving nursing interns’ transfusion-specific theoretical knowledge, procedural skills, and SDL capabilities. Ultimately, this research aims to explore a structured, replicable approach to supplement the traditional rotational internship system, thereby addressing its inherent limitations in delivering high-quality, standardized training for high-stakes specialty nursing procedures.

This study is theoretically underpinned by self-determination theory (SDT) and the BOPPPS instructional design model. SDT posits that intrinsic motivation and SDL are nurtured when three basic psychological needs are satisfied: autonomy (the sense of control over one’s learning), competence (the sense of mastery and achievement), and relatedness (the sense of social connection and belonging in the learning environment). We hypothesize that the BOPPPS model, through its sequential, structured phases, directly addresses each of these core psychological needs: the bridge-in and participatory learning phases foster autonomy by encouraging active student participation and choice; clear, measurable objectives and formative pre/post-assessments build competence by providing explicit learning goals and timely feedback on progress; and collaborative learning activities within the participatory learning phase enhance relatedness among students and between students and instructors. This theoretical alignment provides a robust rationale for selecting SDL as a key primary outcome variable and leads to the hypothesis that a BOPPPS-based blended intervention significantly enhances SDL among nursing interns. A quasi-experimental study design was adopted to test this hypothesized causal relationship, a pragmatic and ethically appropriate approach given the constraints of real-world clinical internship settings—where randomization to training conditions is often logistically unfeasible—while still allowing for rigorous evaluation of educational interventions.

## 2 Materials and methods

### 2.1 Study design and participants

This study adopted a mixed-methods quasi-experimental design to evaluate the effectiveness of a BOPPPS-based blended training model in transfusion nursing education for nursing interns. This design integrated quantitative outcome measures (i.e., pre-and post-intervention theoretical test scores and standardized operational skills assessments) with qualitative exploratory data (semi-structured interviews), underpinned by a pragmatic epistemological approach prioritizing the generation of actionable, practice-oriented insights for clinical nursing education.

Aligned with this pragmatic stance, a prospective, nonrandomized, nonsimultaneous controlled trial framework was used, which was adapted to the authentic constraints and schedule of clinical rotations to ensure ecological validity. The study participants were nursing interns completing their clinical rotation in the Internal Medicine Department of Sichuan Provincial People’s Hospital between October and December 2024. To minimize the risk of intergroup communication and intervention contamination—key threats to internal validity in quasi-experimental designs—a nonsimultaneous control group design was implemented. Specifically, interns who completed their internal medicine rotation from October to November 2024 were assigned to the control group, which received standard traditional transfusion nursing training. In contrast, interns undertaking their rotation in the same department from November to December 2024 were allocated to the experimental group, which participated in the BOPPPS-based blended transfusion nursing training intervention.

The inclusion criteria were as follows: full-time undergraduate nursing students scheduled to complete a 4-week (one-month) internal medicine nursing internship (consistent with the study’s intervention duration) who voluntarily provided written informed consent to participate in the study. The exclusion criteria were as follows: interns who withdrew from the internal medicine internship prematurely, who were scheduled for an extended internal medicine rotation exceeding 4 weeks (to avoid potential cross-group exposure and intervention contamination), or who failed to complete the entire training process (including theoretical learning, simulation practice, and clinical exposure) and all required post-intervention assessments.

### 2.2 Study setting and educational context

This study was conducted at Sichuan Provincial People’s Hospital, a tertiary grade A teaching hospital that serves as a pivotal clinical training base for nursing students in the region. The BOPPPS-based transfusion nursing training module was embedded within the standard four-week internal medicine nursing internship curriculum for participating interns.

#### 2.2.1 Blended learning platforms

Asynchronous learning content—including transfusion nursing theoretical lectures, formative assessments, and supplementary learning resources—was delivered through the Superstar Learning Hub platform (Version 6.7). Synchronous interactive sessions, such as case-based discussions and real-time question and answer sesssions on transfusion-related clinical scenarios, were conducted via Tencent Meeting (Version 3.40).

#### 2.2.2 Simulation training facilities

Hands-on transfusion skills practice was carried out in a dedicated clinical simulation training center. This center was equipped with low-fidelity transfusion arm models (Taigui Medical, Model TG/H6S), mid-fidelity patient manikins (General Practitioner Model, GMH2A), and standard hospital transfusion kits that aligned with clinical practice guidelines.

#### 2.2.3 Clinical exposure

During the third week of the four-week internship, each intern in the experimental group completed one supervised real-world transfusion procedure on a consenting patient. Eligible patients were in a stable clinical condition with uncomplicated vascular access, and all procedures were performed under the direct, one-on-one supervision of a qualified clinical nursing instructor.

### 2.3 Measures to control for confounding variables

We acknowledge that the time-based grouping strategy employed in this study could introduce time-related confounding variables, including the accumulation of teaching team experience over the intervention period and seasonal variations in the volume or complexity of clinical transfusion cases. To mitigate these potential sources of bias and enhance the internal validity of the study, the following targeted control strategies were implemented.

#### 2.3.1 Stabilizing the teaching and assessment team

The same cohort of senior clinical nursing educators was responsible for all instructional delivery, simulation training sessions, and performance assessments for both the control and experimental groups throughout the study period. Prior to the commencement of the study, all participating instructors completed unified standardized training on both the traditional transfusion teaching model and the BOPPPS-based blended model. In addition, the teaching team used identical, pre-developed standardized lesson plans, simulation training scenarios, and structured assessment checklists for all study-related activities. This standardized approach was designed to ensure consistency in teaching content, instructional methods, and evaluation criteria across both groups and over time, thereby minimizing the confounding impact of individual instructor variability or changes in teaching proficiency during the study.

#### 2.3.2 Controlling the clinical practice context

Blood transfusion was selected as the focus of this intervention due to its nature as a highly standardized core nursing procedure with well-delineated, evidence-based procedural steps, which reduces its susceptibility to variability stemming from specific patient disease types or clinical contexts. For simulation-based skills practice, interns in both groups used identical low- and mid-fidelity transfusion training models and standardized hospital transfusion kits. For supervised real-world transfusion procedures, patient cases were systematically selected to ensure comparability between groups in terms of procedural complexity—specifically, the condition of peripheral vascular access—and key clinical indications for transfusion. Furthermore, all live transfusion procedures were performed under direct, one-on-one supervision of trained instructors, who followed standardized clinical scenario guidance to ensure consistency in the practice environment and supervision across groups.

### 2.4 Research eligibility criteria

#### 2.4.1 Sample size calculation

The primary outcome measure of this study was the post-intervention theoretical knowledge test score related to transfusion nursing. Sample size estimation was performed using a formula to compare the means between two independent samples. Drawing on previous research regarding the application of the BOPPPS teaching model in medical education (9), we anticipated a mean difference in post-test scores between the experimental and control groups corresponding to a Cohen’s d effect size of approximately 0.8 (a medium-to-large effect, as defined by Cohen). A two-tailed significance level (*α*) of 0.05 and a statistical power (1-*β*) of 0.80 were prespecified.

The sample size calculation was conducted using G*Power 3.1 software, which yielded a minimum required sample size of 26 participants per group to detect the anticipated effect size. To account for a potential attrition rate of 20% during the internship period (e.g., due to temporary leave, early withdrawal, or incomplete data collection), the target sample size was adjusted to 32 participants per group. Ultimately, 78 participants were enrolled in the study, including 40 in the experimental group and 38 in the control group. This sample size met the minimum statistical requirements for analyzing the primary outcome.

### 2.5 Teaching protocols

#### 2.5.1 Setting and participants

This study was conducted in the Department of Hematology at Sichuan Provincial People’s Hospital. Nursing interns in both the control and experimental groups received instruction on identical core transfusion nursing content and were taught by the same teaching faculty to ensure instructional consistency. The teaching team comprised one head nurse from the Internal Medicine Area, one head nurse from the Hematology Department, two nursing education team leaders, and eight clinical nursing instructors with specialized transfusion nursing expertise.

#### 2.5.2 Teaching protocol for the control group: traditional rotational training

The control group underwent the department’s standard 4-week rotational training program for transfusion nursing, which adopted a conventional instructor-centered didactic teaching approach. This protocol lacked structured online learning components and did not incorporate the BOPPPS instructional framework.

##### 2.5.2.1 Schedule and content

Week 1: A 1-h ward orientation session covering institutional rules, clinical workflow, and basic safety protocols specific to the hematology department.

Weeks 2–3: Two 2-h didactic lectures delivered in accordance with the department’s standard internal medicine nursing syllabus. Blood transfusion was included as one of multiple general internal medicine topics, with content limited to basic transfusion indications and procedural steps; no in-depth case-based discussions or hands-on drills for adverse transfusion reaction management were provided. In addition, one 1-h demonstration of peripheral intravenous catheterization was conducted, but structured hands-on practice of transfusion procedures using simulation models was not included in the training.

Week 4: Completion of departmental standard final assessments for transfusion nursing knowledge and skills.

##### 2.5.2.2 Instructional context

Daily one-on-one clinical mentorship was provided, with a primary focus on routine clinical task supervision. Learning was predominantly passive (e.g., lecture attendance, clinical observation) and opportunistic, dependent on the availability of transfusion-related clinical cases during the rotation. Notably, no formal pre-assessment of baseline knowledge, structured participatory learning sessions, or post-assessment feedback loops were implemented as part of the traditional training protocol.

#### 2.5.3 Teaching protocol for the experimental group: BOPPPS-based blended training model

The experimental group received the same routine 4-week rotational clinical training as the control group, supplemented with a structured BOPPPS-based blended learning intervention tailored to transfusion nursing education. The implementation of this intervention was divided into two sequential phases: precursor preparation and instructional execution.

##### 2.5.3.1 Precursor preparation

###### 2.5.3.1.1 Curriculum enhancement

The nursing education team leaders revised and augmented the standard internal medicine nursing syllabus to develop a specialized transfusion nursing curriculum. This enhanced curriculum expanded content coverage to include detailed transfusion procedural standards, systematic management of adverse transfusion reactions, and case-based scenarios reflective of real-world clinical practice in the hematology department.

###### 2.5.3.1.2 Faculty training and standardization

To ensure uniform and high-fidelity delivery of the BOPPPS-based blended intervention across all instructors, all 12 members of the teaching team completed a standardized prestudy training program consisting of two 4-h interactive workshops covering the following core components:

- Theoretical underpinnings of the BOPPPS model and evidence-based teaching strategies for each phase.
- Technical operation and pedagogical application of blended learning platforms (Superstar Learning Hub for asynchronous content delivery, Tencent Meeting for synchronous interactive sessions).
- Updated clinical transfusion standards, institutional protocols, and patient safety guidelines specific to blood transfusions.
- Hands-on simulation training for transfusion procedures and adverse reaction management using the center’s low- and mid-fidelity models.
- Standardized assessment criteria, rubrics, and feedback protocols for evaluating interns’ knowledge and skills performance.

A unified teaching resource package was distributed to all instructors, which included PowerPoint slides on BOPPPS model theory and its clinical nursing application, a structured workshop agenda and training manual, key reference documents (the hospital transfusion protocol, procedural checklists, and adverse reaction management algorithms), and phase-specific facilitation guidelines to standardize the delivery of each BOPPPS component.

###### 2.5.3.1.3 Faculty competency verification

Following training, all instructors were required to complete a post-training knowledge quiz with a mandated passing score of ≥85% and conduct a mock BOPPPS-based blended teaching session. These mock sessions were evaluated by the Internal Medicine Area head nurse and a nursing education committee member using a standardized observation checklist to verify proficiency in model implementation and ensure adherence to the intervention protocol.

##### 2.5.3.2 Instructional execution (BOPPPS model)

The specialized transfusion nursing training for the experimental group was rigorously implemented in strict adherence to the six sequential phases of the BOPPPS instructional framework. Detailed content regarding the implementation of each phase is presented in Table 1.

**Table 1:** Blended specialty training model based on the BOPPPS model.

Step	Timing	Implementation Content	Teaching method	Activity objectives
Bridge-in (B)	First day of the internship cycle (entry-level education)	Establishment of a dedicated WeChat group for the training; introduction of a clinical transfusion case with adverse reaction scenarios; release of preclass theoretical learning materials (e.g., transfusion guidelines, procedural manuals); formulation and presentation of guiding questions (e.g., key risks in transfusion practice).	Online preclass preparation + offline face-to-face instruction and group discussion	Instructors: Link transfusion nursing content to interns’ prior knowledge and clinical context.
				Interns: Establish an initial connection to transfusion nursing content; develop a preliminary understanding of the transfusion procedure and common adverse reaction types.
Objective (O)	First day of the internship cycle (entry-level education)	Explicit statement of the overall course objectives and training competencies; detailed clarification of key learning points (e.g., transfusion indication judgment) and difficult points (e.g., adverse reaction emergency management); alignment of objectives with clinical practice requirements.	Offline face-to-face teaching	Instructors: Define clear, measurable teaching objectives and prioritize learning content.
				Interns: Comprehend the core content and expected competencies of the training; establish a clear learning roadmap for the internship cycle.
Pre-test (P)	First day of the internship cycle (entry-level education)	Release of an online pre-test consisting of multiple-choice and short-answer questions covering basic transfusion knowledge; assignment of preparatory learning tasks aligned with the participatory learning phase content.	Online standardized assessment	Instructors: Conduct a baseline assessment of interns’ mastery of basic transfusion knowledge; identify knowledge gaps to tailor subsequent teaching content.
				Interns: Self-assess individual proficiency in foundational transfusion knowledge; clarify targeted learning priorities for the training.
Participatory learning (P)*	Internship weeks 1–3 (core training phase)	Systematic theoretical teaching of transfusion nursing (e.g., transfusion compatibility testing, adverse reaction classification); hands-on clinical practice and simulation training (e.g., venous indwelling needle transfusion, adverse reaction emergency drills); case-based group discussions (focused on complex transfusion scenarios and decision-making).	Blended online–offline integration (online asynchronous learning + offline synchronous practice and discussion)	Instructors: Dynamically adjust teaching details based on interns’ performance and feedback; strengthen key and difficult content through interactive learning.
				Interns: Master core transfusion nursing theoretical knowledge and procedural skills; stimulate independent clinical thinking; improve hands-on operational and problem-solving abilities.
Post-test (P)	Upon completion of the core training course	1. Theoretical assessment: Online test focusing on the course’s key and difficult points (e.g., adverse reaction management protocols, transfusion safety standards); 2. Operational assessment: Offline practical evaluation based on the hospital’s Venous Indwelling Needle Blood Transfusion Technology Operation Process and Evaluation Criteria.	Online theoretical test + offline standardized operational assessment	Instructors: Evaluate the achievement of predefined teaching objectives; collect assessment data to provide evidence to optimize the training program.
				Interns: Consolidate and refine transfusion-related theoretical knowledge and operational skills; achieve proficiency in clinical transfusion practice competencies.
Summary (S)	Last week of the internship cycle (exit evaluation phase)	Facilitated faculty–student group discussions for training content review and summary; administration of online anonymous teaching evaluation questionnaires; conducting of semi-structured individual interviews with interns.	Offline face-to-face discussions + online anonymous evaluation	Instructors: Collect comprehensive feedback from interns on the training model and content; identify strengths and deficiencies to inform subsequent teaching program revisions.
				Interns: Reinforce mastery of training content and learning objectives; reflect on the learning process; enhance independent thinking ability and learning initiative.

*Participatory Learning: The primary objective of this participatory learning phase was to engage nursing interns in structured, progressive hands-on practice to facilitate active knowledge construction and transfusion skills mastery. The clinical practice component was implemented during Weeks 1–3 of the internship. Considering patient safety and the initial competency level of interns in the early stages of clinical rotations, the practice was designed in adherence to the principle of “from simulation to real-world application, and from decomposed skill training to integrated procedural practice”.

Practice Schedule and Duration: Offline hands-on training for transfusion procedures was conducted once weekly, with each session lasting approximately 10 min. Over the entire intervention period, each intern accumulated approximately 30 min of hands-on practice on the simulation models. In addition, during Week 3, each nursing intern performed one supervised real-world transfusion procedure on a consenting patient under direct instructor supervision. This phased schedule was tailored to familiarize interns progressively with the core steps of transfusion procedures while ensuring patient safety.

Instructional methods: A standardized “demonstration–imitation–feedback” teaching cycle was adopted throughout the practice phase. First, the supervising instructor conducted a step-by-step demonstration of the transfusion procedure, with concurrent explanations of key operational points and safety precautions. The interns then practiced the procedure on simulation models, with an instructional focus placed on ensuring “procedural accuracy and standardization.” Feedback was predominantly provided in real time, focusing on immediate error correction and the reinforcement of correct techniques. During the Week 3 real-world procedure, the instructor maintained “close, one-on-one supervision” to mitigate potential risks and ensure patient safety. A brief debriefing session was conducted immediately after each live procedure to review performance, address issues, and consolidate learning.

Simulation fidelity: Low-fidelity transfusion simulators (Taigui Medical, Model TG/H6S) were used for practice in Weeks 1 and 2. These models are well-suited for training in correct procedural sequencing and instrument handling but do not provide the tactile feedback of real venipuncture or simulate physiological patient responses to transfusion. In contrast, the supervised real-world transfusion procedure in Week 3 offered a high-fidelity clinical scenario, allowing interns to apply learned skills in an authentic clinical context with actual patient interactions and physiological feedback.

## 3 Mixed-methods design and qualitative component

### 3.1 Overall design and integration rationale

**Figure 1:**
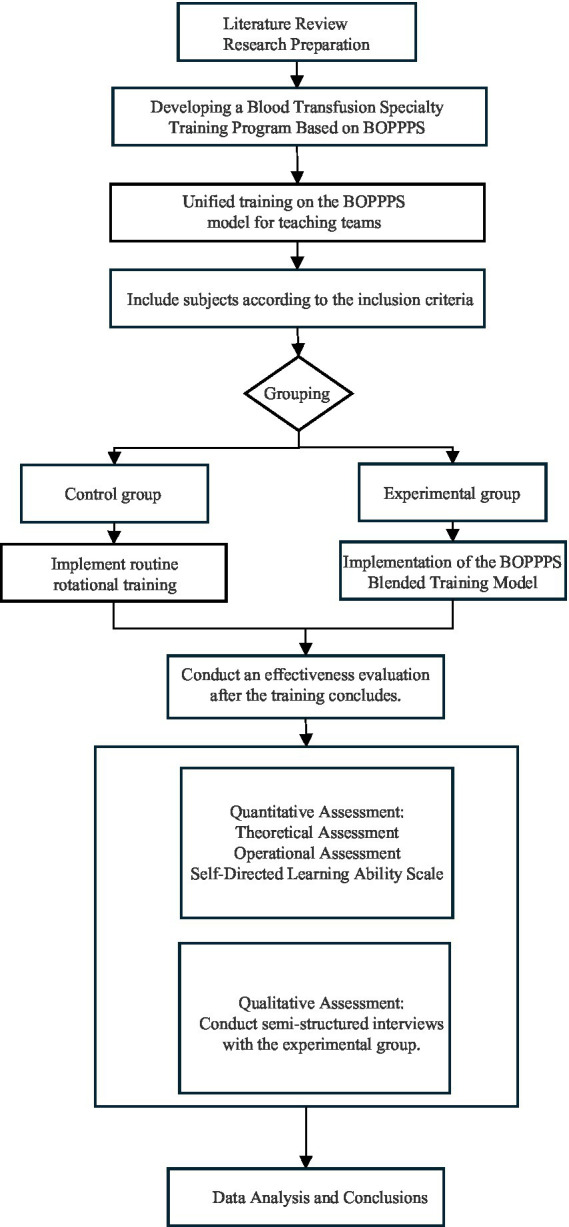
Technology roadmap.

Based on the theoretical framework linking the BOPPPS model to SDT, this study adopted an explanatory sequential mixed-methods design. Quantitative data were first collected and analyzed, followed by qualitative semi-structured interviews with the experimental group only. The qualitative phase aimed to contextualize and explain quantitative outcomes, particularly exploring the mechanisms by which the BOPPPS model influenced learning processes and enhanced SDL. Figure 1 depicts the technical roadmap of this study, outlining the overall methodological framework, including participant grouping, implementation of different training models and the sequential integration process of quantitative and qualitative analyses.

### 3.2 Qualitative data collection and analysis

#### 3.2.1 Sampling and procedure

Post-training semi-structured interviews were conducted with a purposive sample of experimental-group interns until thematic saturation (*n* = 13). These interviews were performed by a qualitative research–trained investigator, who maintained a reflexive journal to bracket preconceptions.

#### 3.2.2 Rigor and trustworthiness

To ensure credibility, two researchers independently coded three transcripts, achieving excellent intercoder reliability (Cohen’s kappa = 0.85). Member checking was conducted with three participants, and an audit trail was maintained. The interview guide (Supplementary File 1) used neutral phrasing (e.g., “describe and compare your experience”).

#### 3.2.3 Analysis

Interviews were transcribed verbatim and analyzed via NVivo 20 using Colaizzi’s phenomenological method (10). The derived themes were validated through member checking.

### 3.3 Evaluation indicators and variables

#### 3.3.1 Independent variable

##### 3.3.1.1 Training model

Participants were assigned to either the BOPPPS-based blended training (experimental group) or traditional rotational training (control group).

#### 3.3.2 Dependent variables

1. Theoretical Knowledge Score

Operational Definition: A proctored written exam (full score = 100) administered in Week 4 that assessed five core transfusion competencies: indications, procedures, adverse reaction identification, management, and case application.

Composition: Composite score of multiple-choice questions (40%), short-answer questions (30%), and case analyses (30%). An exam blueprint ensured content validity across all competencies.

1. Operational skills score

Operational Definition: Evaluated via the hospital’s mandatory Intravenous Catheter Blood Transfusion Operational Procedures and Quality Standards checklist (full score = 100) in a simulated setting post-rotation.

Components: Four weighted domains—pretransfusion verification/preparation (15 points), aseptic technique/puncture skills (50 points), procedural quality (25 points), and knowledge application (10 points). All assessments were conducted by two precalibrated trained assessors.

1. Self-directed learning capacity score

Operational Definition: Measured using the 28-item Nursing College Students’ Independent Learning Ability Scale (11), administered on a five-point Likert scale, with total scores ranging from 28 to 140 (higher scores indicate better SDL capacity). This scale includes three subscales: self-management, information seeking, and collaborative learning.

Administration: Completed at baseline (rotation entry) and post-intervention (rotation exit).

#### 3.3.3 Covariates

Baseline characteristics (age, sex) and pre-rotation theoretical scores (same exam as post-test, administered at baseline) were collected as covariates to control for confounding in the comparative analyses.

### 3.4 Data analysis

Data analysis followed an explanatory sequential design.

#### 3.4.1 Quantitative analysis

Analyses were performed using IBM SPSS 25.0. Continuous data are presented as mean ± SD, with between-group comparisons via independent-samples *t*-tests and within-group comparisons (for SDL) via paired-samples *t*-tests. Categorical data were compared using *χ*^2^ tests. Statistical significance was set at two-tailed *α* = 0.05.

#### 3.4.2 Qualitative analysis

As detailed in Section 3.2, interview transcripts were analyzed via NVivo 20 using Colaizzi’s phenomenological method.

#### 3.4.3 Integration

The quantitative results identified changes, and the qualitative themes explained the mechanisms (how/why these changes occurred). A full synthesis of the quantitative and qualitative findings is presented in the Discussion section.

## 4 Results

### 4.1 Quantitative data results

A total of 81 nursing interns were enrolled in this study, including 43 in the experimental group and 38 in the control group. Three interns in the experimental group were excluded due to incomplete training, resulting in a final sample of 40 in the experimental group and 38 in the control group.

Comparisons of the general characteristics between the two groups are presented in Table 2.

**Table 2:** Comparison of baseline demographics and theoretical and operational scores between groups $\;(\bar{x}$ ± s).

Group	Number	Sex		Age	Theoretical knowledge	Operational performance
		Male	Female			
Experimental group	40	5	35	20.95	68.91 ± 10.53	90.35 ± 2.02
Control group	38	4	34	21.03	60.47 ± 17.04	89.71 ± 1.94
Statistic		*χ*^2^ = 0.74		*t* = −0.213	t *=* 2.645	t *=* 1.419
*p*-value		0.785		0.482	0.010	0.160

Comparisons of SDL capacity and readiness for independent learning between the two groups of nursing interns before and after the intervention are presented in Table 3.

**Table 3:** Comparison of self-directed learning between groups of nursing students before and after teaching $\;(\bar{x}$ ± s).

Groups	Quorum	Pre-test				post-test			
		Self-management	Information seeking	Collaborative learning	Total score	Self-management	information seeking	Collaborative learning	Total score
Experimental group	40	31.85 ± 4.07	36.68 ± 4.61	23.43 ± 2.94	91.95 ± 8.24	37.45 ± 3.53	39.58 ± 5.25	28.85 ± 3.51	105.88 ± 7.30
Control group	38	32.29 ± 4.74	36.29 ± 4.18	22.34 ± 2.67	90.92 ± 8.08	33.66 ± 4.09	34.34 ± 3.60	26.84 ± 4.46	94.84 ± 8.17
*t*-value		−0.440	0.386	1.698	0.557	4.387	5.108	2.202	6.278
*p*-value		0.661	0.700	0.094	0.579	<0.001	<0.001	0.031	<0.001

### 4.2 Qualitative interview results

A total of 27 nursing interns in the experimental group volunteered for the interviews. Data saturation was achieved after 13 participants had been interviewed, and thematic coding of the transcripts yielded three primary themes and nine secondary themes, which are structured, numbered, and presented in Table 4.

**Table 4:** Thematic coding results of qualitative interviews with experimental group interns.

Primary theme no.	Primary theme	Secondary theme no.	Secondary theme	Key findings and representative quotes
1	Identified deficiencies in clinical specialty practice	1.1	Inadequate clinical exposure to blood transfusion practices	Over half of the interviewees reported limited hands-on transfusion experience.
				P2: “Prior to this training, I had attended lectures on only blood transfusion; actual procedures were always performed by instructors, and I never got to practice myself.”
				P4: “We were only assigned to ward rounds and never allowed to perform transfusions—teachers were worried about potential errors.”
				P7: “I had no exposure to blood transfusion procedures whatsoever before this training.”
		1.2	Limited opportunities for specialty nursing practice	Interns’ duties were dominated by basic tasks (e.g., morning care, vital sign measurement), with minimal specialty practice opportunities.
				P1: “General wards have us doing trivial work; in contrast, ICUs (with no family presence) allow interns to participate in more procedures and learn more skills.”
				P5: “For pediatric infusion—where children’s veins are hard to locate—nursing interns are never allowed to perform the procedure.”
				P6: “Every day is just checking blood pressure and blood sugar. I already know how to do these tasks, and I feel like I have not learned anything new.”
2	Suggestions for optimizing the training model	2.1	Appropriately designed curriculum difficulty	Curriculum aligned with national standards and basic nursing knowledge; all interviewees rated difficulty as moderate and content as focused.
				P1: “The difficulty level was just right, matching the knowledge taught in school.”
				P5: “The difficulty was appropriate—it did not exceed the syllabus and does not need to be increased.”
				P10: “Key content was highlighted, making knowledge clear and easy to master.”
		2.2	Excessive participant numbers in practical training sessions	Interviewees suggested reducing group size to improve the visibility of demonstrations.
				P3: “Too many people attend each practical session; sometimes it’s hard to even see the instructor’s demonstration.”
				P4: “Crowds gather around the demonstration area, making it impossible to observe clearly. The patients also seem nervous, with so many people watching.”
				P7: “Current sessions are too crowded. Instead of one large group, we could extend training time and split it into small groups of 4–6 people. This way, everyone can see the demonstration clearly.”
		2.3	Complementary advantages of online and offline learning formats	Blended format recognized as beneficial; most preferred offline learning for better outcomes.
				P2: “I do not particularly like offline sessions, as they delay my return home, but I have to admit that offline learning is more effective.”
				P5: “If theoretical content is delivered online, students may not pay attention. Explaining theory while demonstrating operations is much more helpful than tedious lectures.”
				P8: “Offline training sessions run a bit long—we start after work and finish at 7 p.m. However, online materials may not be viewed by students at all.”
3	Perceived gains and value of specialty training	3.1	High acceptability of the BOPPPS model	All interviewees expressed positive acceptance of the model and reported learning benefits.
				P4: “I find the BOPPPS model very acceptable. The pre-test helps identify my knowledge gapsand deepens my understanding of the operational steps. I also think sharing lecture PPTs in advance via group chat is a good idea.”
				P8: “This training model is more memorable than traditional lectures—it should be used more often!”
				P11: “The model is less boring than conventional learning methods, so it leaves a much stronger impression.”
				P13: “I previously attended full-day lecture-based training in the pediatric department. The instructor covered too much content too quickly, with no hands-on practice. This BOPPPS training, with its practical components, is far more impressive.”
		3.2	Strong demand for specialty training	Interviewees emphasized specialty training as essential to compensate for limited clinical practice opportunities.
				P6: “Specialty training is definitely necessary. We never get exposed to specialty knowledge in clinical settings; teachers do not let us practice these skills. Every day is just wiping tables and checking blood pressure.”
				P7: “Specialty training is a must. Most departmental training consists of PowerPoint lectures with no hands-on practice. We need opportunities to actually perform specialty procedures.”
				P10: “I hope instructors can teach us knowledge and skills that we have not been exposed to before—I want to learn something new.”
		3.3	Knowledge and skills gains from blood transfusion training	Nursing interns reported improved understanding of transfusion procedures and adverse reaction identification.
				P1: “First, the training consolidated my existing blood transfusion knowledge and deepened my understanding of transfusion reactions. Second, hands-on practice and mentorship made the procedures more concrete. I will be much more confident when performing transfusion-related tasks in the future.”
				P5: “I now have a clear understanding of the blood transfusion process. Before the training, I knew only the basics and had never performed a transfusion myself.”
				P7: “I can now clearly categorize transfusion reactions and outline the full transfusion process. Previously, I just watched instructors perform transfusions, without understanding the complete workflow.”
				P11: “Before this training, I only grasped about 20% of blood transfusion knowledge. Now, I feel I have mastered around 60% of the content.”
		3.4	Desired Areas for Future Specialty Training	Nursing interns expressed strong willingness for training in unexposed specialty areas: five for PICC, three for stoma care, two for dialysis.
				P4: “I want to learn about ventilators. I heard about them during my ICU rotation, but we were never allowed to adjust parameters or practice using them.”
				P5: “Procedures like closed chest drainage in ICU and surgery departments seem different from textbook descriptions—I want to learn these skills.”
				P10: “I’m interested in any hands-on training, except for IV infusion—we already get plenty of practice with that in inpatient wards.”

## 5 Discussion

### 5.1 Explaining BOPPPS efficacy disparity: knowledge gains vs. skills assessment outcomes

Our study adopted a blended learning model integrating online theoretical modules with offline, BOPPPS-structured simulation sessions. This design aimed to synergize the flexibility of digital learning with the interactive depth of the BOPPPS framework. The significant improvement in theoretical scores not only validates the well-established efficacy of BOPPPS in medical education (12–14) but also clarifies its underpinning cognitive mechanisms, which are grounded in established learning theories.

The structured BOPPPS cycle facilitates deeper information processing and active knowledge construction, with its design theoretically grounded in established learning frameworks. Specifically, the pre-assessment phase aligns with cognitive load theory, efficiently identifying prior knowledge gaps to enable the targeted allocation of instructional resources (15). The participatory learning phase then operationalizes constructivist learning theory, engaging interns in authentic problem-solving and collaborative discourse to build and refine cognitive schemata (16). This theory-driven active learning cycle fosters superior knowledge retention compared to passive didactic methods.

Regarding skills acquisition, the nonsignificant between-group difference necessitates nuanced analysis, which we explain through two key factors related to practice design and assessment tools. First, the practical component of this study was constrained by its limited duration and reliance on low-fidelity models for decomposed skill drills. While BOPPPS effectively builds procedural understanding (clarifying the why and when), translating this into automated procedural fluency depends heavily on high-intensity, high-fidelity practice, and quality feedback—elements limited in this initial implementation (17). Second, the standardized hospital checklist used for skills assessment primarily certifies basic safety and psychomotor correctness and lacks the sensitivity to detect the nuanced clinical judgment and adaptive decision-making that BOPPPS aims to develop. Thus, the absence of a score difference does not negate improvements in these higher-order cognitive aspects of skills.

In conclusion, our findings refine the optimal application of BOPPPS in skills-based training. Future designs should pair the model’s cognitive scaffolding with robust, high-fidelity practice ecosystems and complementary assessments that capture clinical reasoning capabilities.

### 5.2 BOPPPS-driven enhancement of self-directed learning: mechanisms, evidence, and future directions

A pivotal finding of our study was the significant improvement in SDL scores among the experimental group. We attribute this outcome to the BOPPPS model’s inherent capacity to satisfy the core psychological needs delineated in SDT (18), thereby fostering intrinsic motivation. Specifically, the bridge-in phase establishes clinical relevance, and the participatory learning phase grants intern’s agency in problem-solving—both directly nurturing a sense of autonomy. Clear learning objectives and pre-assessments provide a transparent roadmap and personalized feedback, enabling interns to track progress and address knowledge gaps systematically, which builds a tangible sense of competence. Furthermore, instructor-facilitated collaborative activities in the participatory learning phase create a supportive community that fulfills the need for relatedness (19). By addressing autonomy, competence, and relatedness in tandem, the BOPPPS model transforms learning from a passive obligation into an internally driven inquiry.

This theoretical mechanism was strongly corroborated by our qualitative findings. Interns’ reports of “sparked interest in exploring other specialties” (Theme 3.4) and their positive feedback on the model’s clarity and hands-on opportunities (Themes 3.1, 3.2) serve as concrete evidence of enhanced autonomy and competence, directly explaining the quantitative gains in SDL scores.

A key limitation of this study is the lack of qualitative feedback from the control group regarding how traditional teaching influences psychological needs. Nevertheless, our findings align well with the established educational literature, which indicates that teacher-centered didactic instruction often fails to satisfy these fundamental needs due to unclear goal relevance, limited student choice, and delayed feedback—factors that tend to promote passive learning (20–22). Thus, the increased learning initiative observed in the experimental group (e.g., interest in exploring new specialties) can reasonably be attributed to the BOPPPS model’s ability to mitigate the motivational shortcomings of conventional teaching.

Another limitation is the absence of longitudinal follow-up to assess the sustainability of SDL improvements or their transfer to subsequent clinical rotations—a critical direction for future research. Theoretically, intrinsic motivation cultivated by satisfying basic psychological needs is a more stable driver of long-term behavioral change than extrinsic rewards (23); therefore, the significant post-intervention SDL gains reported here provide a promising theoretical and empirical basis for hypothesizing durable effects. To confirm whether the BOPPPS model instills a lasting lifelong learning habitus, future studies should adopt a longitudinal design, reassessing SDL at intervals (e.g., 1, 3, and 6 months post-training) and complementing scale data with objective behavioral metrics (e.g., frequency of independent knowledge seeking and quality of clinical questions posed) during later practice.

In conclusion, the BOPPPS model functions not only as a tool for knowledge delivery but also as a robust framework for cultivating metacognitive skills and lifelong learning attitudes—competencies that are indispensable in the rapidly evolving healthcare landscape (24, 25).

### 5.3 Broader implications, limitations, and future research directions

Beyond immediate efficacy in hematology transfusion training, this study illustrates how a structured BOPPPS-blended approach may address longstanding challenges in nursing education, such as limited hands-on practice opportunities and inconsistent clinical supervision (26). Notably, our findings prompt critical reflection on current procedural assessment paradigms: while standardized checklists are indispensable for safety validation, they may fail to capture the nuanced clinical judgment and adaptive reasoning that the BOPPPS model aims to foster. Thus, this intervention serves as a promising, structured prototype for high-stakes specialty training, with its well-documented design and positive outcomes providing a clear blueprint for adaptation and pilot testing in other clinical domains.

To fully evaluate broader potential, future research should pursue two parallel priorities: (1) curriculum development, creating BOPPPS-based modules for other high-risk, low-frequency clinical procedures, and (2) assessment evolution, designing tools sensitive to the higher-order cognitive gains induced by this model. In summary, this study establishes the BOPPPS-blended model as an effective educational strategy for transfusion training. Translating this localized success into a generalizable, competency-assured framework will require longitudinal and multicenter studies to verify its implementation fidelity, cost-effectiveness, and efficacy across diverse specialties and institutional settings.

These future directions are directly informed by the present study’s limitations. As a single-center trial with a modest, homogeneous sample from one clinical setting, its generalizability is constrained. Despite efforts to control confounding in the nonrandomized design, residual time-related bias cannot be ruled out. Therefore, future research should leverage multicenter collaborations with larger, more heterogeneous samples—including interns from different hospital tiers and educational backgrounds—to rigorously test the model’s applicability. Where randomization is impractical, methodological rigor can be strengthened through detailed reporting of period comparability (e.g., case mix, instructor workload) and sensitivity analyses to quantify the impact of unmeasured confounders. Through such endeavors, a more adaptive, competency-guaranteeing educational ecosystem can be built—one that equips nurses not only to perform established procedures but also to navigate the uncertainties of future clinical practice.

## Data Availability

The raw data supporting the conclusions of this article will be made available by the authors, without undue reservation.
