# Supplementary material for: Innovating transfusion training: a BOPPPS-based blended learning model to enhance nursing interns’ specialized competency
Source: Front Med (Lausanne). 2026 Feb 10;13:1759418. doi: 10.3389/fmed.2026.1759418 (PMC12929102; doi:10.3389/fmed.2026.1759418)
Supplement: Supplementary file 1 [file Supplementary_file_1.pdf]

Supplementary File 1: Semi-structured Interview Guide

Study Title: Innovating Transfusion Training: A BOPPPS-Blended Learning Model to Enhance Nursing Interns' Specialized Competency

Purpose: This guide was used to conduct in-depth, semi-structured interviews with nursing interns in the experimental group following the BOPPPS-based blended transfusion training. The aim was to neutrally explore their subjective experiences, perceived learning processes, challenges, and suggestions.

Interview Context: Conducted post-training in a private setting. Each interview lasted approximately 20 minutes, was audio-recorded with consent, and transcribed verbatim.

Opening Script (Read by Interviewer):

"Thank you for your participation. This interview aims to understand your personal experience with the transfusion training you just completed. There are no right or wrong answers; we value your honest perspective. Our conversation will be audio-recorded for accuracy, and all information will be kept strictly confidential and anonymous. Do you have any questions before we begin?"

Core Interview Questions & Prompts

Part I: Experience with the Training Model & Structure

1.General Experience: "Walk me through your experience with this transfusion training course. What stood out to you?"

Probe (if needed): "How did you find the arrangement of content and the highlighted key points?"

2.Blended Learning Format: "Can you describe your experience with the mix of online and offline learning activities? What aspects worked well for you, and what

aspects were challenging?”

Probe (if needed): “How did this format compare to your expectations or to other learning experiences?”

3.BOPPPS Teaching Structure: “The training used a specific structure (pre-test, teaching, practice, post-test). What was it like for you to learn within this structure?”

Probe (if needed): “How does this structured approach compare to the way you typically learn during clinical rotations?”

Part II: Perceived Learning Outcomes and Application

4. Clinical Application: “Thinking about applying this in a real clinical setting, how do you now approach or think about the process of blood transfusion and monitoring for reactions?”

Probe (if needed): “What feels more manageable, and what might still feel uncertain or challenging?”

5. Overall Reflection & Suggestions: “Looking back, what would you say are the main takeaways for you from this training? This could be about knowledge, skills, or even how you learn.”

Probe (if needed): “Based on your experience, what specific changes would you suggest to improve this training for future interns?”

6. Future Directions: “In what contexts or for which other nursing skills do you think a training approach like this would be most valuable or needed, and why?”

Closing Script (Read by Interviewer):

“These are all my planned questions. Is there anything important about your training experience that we haven’t discussed? Thank you again for sharing your time and insights.”
